# Social stress in adolescents induces depression and brain-region-specific modulation of the transcription factor MAX

**DOI:** 10.1038/tp.2016.202

**Published:** 2016-10-11

**Authors:** L S Resende, C E Amaral, R B S Soares, A S Alves, L Alves-dos-Santos, L R G Britto, S Chiavegatto

**Affiliations:** 1 Department of Pharmacology, Biomedical Sciences Institute, University of Sao Paulo, Sao Paulo, Brazil; 2 National Institute for Developmental Psychiatry (INCT-CNPq), Department of Psychiatry, Institute of Psychiatry, University of Sao Paulo Medical School, Sao Paulo, Brazil; 3 Department of Physiology and Biophysics, Biomedical Sciences Institute, University of Sao Paulo, Sao Paulo, Brazil

## Abstract

MAX is a conserved constitutive small phosphoprotein from a network of transcription factors that are extensively studied in tumorigenesis and whose functions affect cell proliferation, differentiation and death. Inspired by its higher expression during development and in regions involved in emotional behaviors, we hypothesized its involvement in cerebral changes caused by early-life stress. We studied the effects of repeated social stress during adolescence on behaviors and on MAX and its putative partner MYC. Thirty-day-old C57BL/6 male mice underwent brief daily social defeat stress from an adult aggressor for 21 days. Following social stress episodes and housing in social groups after each defeat, adolescent mice exhibit depressive-like, but not anxiety-like behaviors and show higher MAX nuclear immunoreactivity in hippocampal (HC) but not prefrontal cortical (PFC) neurons. Conversely, MAX immunoreactivity is lower in the striatum (ST) of defeated adolescents. The positive correlation between MAX and MYC levels in the PFC revealed disruptions in both the HC and ST. The changes in MAX protein levels are not due to differential gene expression or protein degradation in those regions, suggesting that posttranscriptional modifications occurred. These findings indicate that repeated, brief social defeat in adolescent male mice, combined with group housing, is a useful protocol to study a subtype of depression that is dissociated from generalized (non-social) anxiety. To our knowledge, this is the first report of an association between dysregulation of the MAX-MYC network in the brain and a behavior, suggesting a novel approach for exploiting the neuroplasticity associated with depression.

## Introduction

Early life constitutes a particularly sensitive period during which chronic stress may lead to dysregulation of the stress system, thereby compromising neurodevelopment.^1^ Psychological and experiential factors are among the most powerful stressors.^2^ Approximately 10–30% of children and adolescents, more boys than girls, regularly suffer from school bullying worldwide.^3, 4^ The adverse experience of being bullied by peers induces various potential short- and long-term psychological and somatic sequelae.^5^ Therefore, bullying is considered a risk factor for various mental disorders among adolescents.^6^

Social conflict models in rodents produce several behavioral and physiological changes resembling the symptoms observed in humans, many of which can also be long-lasting.^7, 8, 9, 10^ Repeated social defeat is a valuable animal model for bullying in humans, but most studies have been performed in adult animals. Little is known about how the adolescent brain responds to social defeat.^11^

Because social defeat stress in adolescents perturbs neurodevelopment, we chose to investigate a transcription factor essential to events occurring during this phase and whose expression is higher in regions involved in emotional behaviors. MAX is a highly conserved small phosphoprotein belonging to a network of transcription factors whose interactions result in gene-specific transcriptional activation or repression.^12^

The loss of MAX function in mice is lethal, suggesting an essential role in early embryonic growth and development.^13^ The steady-state expression of *Max* mRNA was temporally investigated in the central nervous system of male mice. The neonatal mouse cerebrum shows high *Max* expression with a prominent reduction during the first 7 weeks of age, when it is stably maintained at lower levels.^14^ This observation is consistent with roles for the MAX network in cell proliferation, differentiation and death, which are important events during neurodevelopment.^15^ Our hypothesis that MAX participates in the cerebral changes caused by early-life stress is further supported by two distinct lines of *in vivo* evidence. *Max* is differentially expressed in the medial prefrontal cortex (PFC) of rats from lines that were bidirectionally selected for ethanol preference^16^ and is upregulated in the FC of rats susceptible to behavioral deficits after an acute unavoidable stress.^17^ Although these studies used outbred adult rats under different conditions and stimuli, to our knowledge, these are the only findings relating the brain regulation of MAX to rodent behavior, which remains otherwise unexplored.

## Materials and methods

### Animals

Male C57BL/6 mice were weaned at 21 days old and group-housed in standard cages. Adult male CD-1 mice were single-housed for 3 weeks and served as the stimulus (residents) in agonistic encounters. They were previously selected for their reliability in shorter attack latencies. All animals had free access to food and water on a 12-h light/dark cycle (on at 0700 hours) in closed, ventilated stands. The experiments followed international guidelines and were approved by local committees.

### Repeated brief social stress

**Figure 1:**
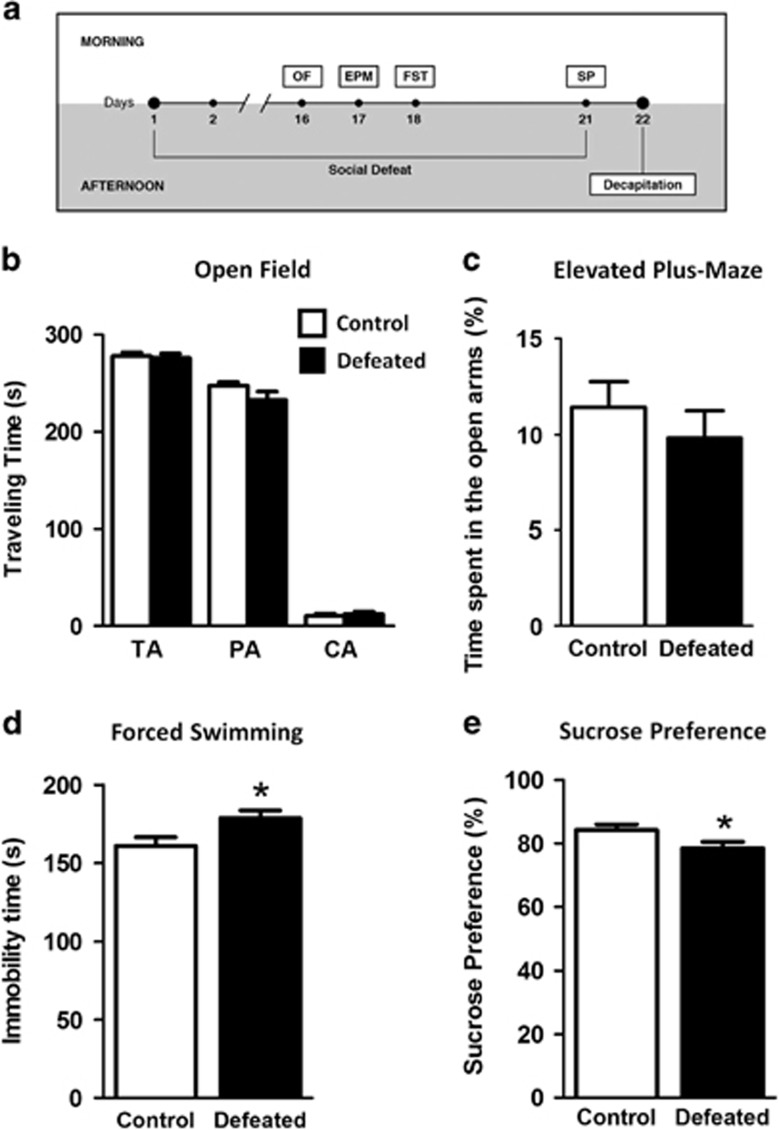
Experimental design and effects of repeated episodes of social defeat on the behaviors of male adolescent mice. (**a**) Thirty-day-old mice were socially defeated for a period of up to 5 min of physical interaction followed by 30 min of cohabitation with the aggressor (threat period) daily during the afternoon for 21 days. The animals were submitted to the open field (OF), elevated plus-maze (EPM), and forced swimming (FS) tests during the morning and to the sucrose preference (SP) overnight. (**b**) OF: travelling time (s) in the total area (TA), peripheral area (PA) and central area (CA) over 5 min. (**c**) EPM: % time spent in the open arms during 5 min. (**d**) FS: time spent immobile (s) in the final 4 min. (**e**) SP: %=sucrose intake (ml)/total fluid intake (ml) × 100. Means±s.e.m.; *P<0.05; *n*=15–18 each for OF, FST, SP; *n*=6 each for EPM.

Social stress was performed according to Miczek *et al.*^18^ for adult mice, with some modifications. Each 30-day-old mouse was introduced into the home cage of an unfamiliar male CD-1 daily afternoon for 21 consecutive days. The residents were rotated every day to maintain the intensity level of each defeat. During the agonistic interactions, the experimental mouse was repetitively attacked by the adult mouse (short bursts of approaching, chasing and biting). As soon as they displayed a clear and sustained submissive posture (vocalization, defensive upright and freezing) they were separated by a wire mesh introduced into the center of the cage, never exceeding 5 min. This condition was maintained for 30 min. Using this procedure, the experimental mice were protected from repeated attacks but remained in olfactory, visual, and auditory contact with the aggressor (threat), which is highly stressful.^19^ Each control animal was concomitantly placed in cages under similar conditions, but without the resident, in a different room. The animals were then relocated to their original cages. We performed independent sets of social stress, followed by behavioral tests (see Figure 1 for timing details).

The open-field (OF), forced swimming (FS) and sucrose preference (SP) tests were recorded from set 1 (*n*=15 control and *n*=18 defeated) and the elevated plus-maze (EPM) from set 2 (*n*=6/6). Blood corticosterone (CORT) and gene expression levels were determined from set 3 (*n*=6/6). Immunoblotting was performed using set 4 (*n*=6/9), immunohistochemistry and immunofluorescence were performed using set 5 (*n*=5/5) and proteasome activity was determined using set 6 (*n*=5/5).

### Behavioral studies

Animals from both groups were tested in an alternating sequence from 0800 hours to 1200 hours The sessions were recorded, and the images were subsequently analyzed using ViewPoint (Videotrack 3.0; View Point, Lyon, France) by researchers blinded to the experimental groups.

#### OF

This test was performed as described.^20^ The apparatus consisted of a square arena (60 cm) covered with white Formica divided into 36 equal squares, with 30-cm-high walls. The animals were tested over a 5-min period in an illuminated room (62 lux at arena floor). The following parameters were considered: total distance traveled, total travel time and time spent in the peripheral area or in the 16 center quadrants.

#### EPM

This test was performed as described.^20^ The apparatus was made of black Formica with two opposing open arms (30 cm × 5 cm; 82 lux) and two closed arms (15 cm high; 39 lux) of the same size that extended from a central platform (69 lux) elevated 47 cm above the floor. Each mouse was placed on the central area with the head facing an open arm and allowed to explore the maze for 5 min. The time spent in both types of arms was quantified.

#### FS

This test was performed according to Ambar and Chiavegatto.^21^ Each mouse was placed in a glass cylinder (17 cm diameter × 24 cm depth) containing 19 cm of 25 °C water for 6 min. Fresh water was used for each animal. The duration of immobility was analyzed during the last 4 min.

#### SP

This test was adapted from a published protocol.^22^ The animals were caged individually after the eighteenth day of social confrontation and were habituated with two identical plastic bottles filled with water and placed on the cage lid for 24 h. The next day, one bottle was replaced with a bottle containing 2% sucrose. This procedure was repeated for one additional day with a fresh sucrose solution, and the relative position of the bottles (left vs right) was changed every 12 h. At the end of the twenty-first day, the amount consumed by each mouse over 48 h was quantified. SP was calculated as sucrose intake (ml)/total fluid intake (ml) × 100.

### Brain and blood samples

Twenty-four hours after the last social confrontation, the animals were decapitated, and the PFC, hippocampus (HC) and striatum (ST) were dissected from both hemispheres on dry ice and stored at −70 °C. Samples of trunk blood were centrifuged (10 000 *g*, 15 min, 4°C), and serum was stored at −70 °C. For immunohistochemistry, mice were intraperitoneally anesthetized with ketamine:xylazine (5:1 mg per 100 g ml^−1^) and intracardially perfused with PBS at 37 °C and 4% paraformaldehyde in cold 0.1 M PB, pH 7.4. Brains were fixed in 4% paraformaldehyde overnight and stored in a cryoprotective 30% buffered sucrose solution.

### Blood corticosterone

CORT was measured in triplicate using an enzyme immunoassay (DetectX Corticosterone Kit, K014-H5, Ann Arbor, MI, USA). Serum samples were diluted 1:100 in assay buffer to ensure that they were within the range of the calibration curve. The sensitivity of the kit was 18.6 pg ml^−1^.

### Immunoblotting

Total protein was isolated as previously described.^23^ Twenty microgram of total protein for MAX and 10 μg for MYC were separated by electrophoresis on 10% Mini-Protean gels (Bio-Rad, Hercules, CA, USA) and transferred to polyvinylidene difluoride (PVDF) membranes using a Semi-dry Trans-Blot System (Bio-Rad). The proteins were blocked (Tris-buffered saline with Tween 20 and 5% bovine serum albumin for 4 h at room temperature (RT)) and incubated overnight at 4°C with a MAX antibody (1:2000; sc-197, Santa Cruz Biotechnology, Santa Cruz, CA, USA)^24^ or a MYC antibody (1:1000; sc-40).^25^ The blots were then incubated with secondary antibodies (1:4000; sc-2004 or sc-2005, 1 h at RT). For the loading control, the membranes were stripped with 5% acetic acid for 5 min at RT, blocked with 5% bovine serum albumin for 1 h and incubated with anti-β-Actin (1:1000; sc-47778) or anti-γ-tubulin (1:10 000; T6557, Sigma-Aldrich, St. Lewis, WA, USA) antibodies overnight at 4 °C. The protein levels were detected with enhanced chemiluminescence (Bio-Rad) and analyzed with ImageQuant 7.0 (GE Healthcare Life Sciences, Piscataway, NJ, USA). The levels of both loading control proteins (β-actin and γ-tubulin) were similar between groups. The optical densities of the MAX and MYC bands were determined by normalization to the corresponding control bands.

### Immunohistochemistry and immunofluorescence

Immunolabeling was conducted as described previously.^26^ Brain coronal sections (30 μm) were incubated with the MAX antibody described above (1:200 in PB, 0.3% Triton X-100, 5% normal goat serum) for 12 h at RT. The sections were incubated with a biotinylated IgG (1:200; Jackson Labs, West Grove, PA, USA) for 2 h, processed for 90 min with the ABC Elite kit (Vector Labs, Burlingame, CA, USA), and visualized using peroxidase (0.05% DAB and 0.03% hydrogen peroxide in PB). The sections were mounted on glass slides, the staining was intensified (0.05% osmium tetroxide in water), and then the sections were dehydrated and coverslipped with Permount (Fisher Scientific, Waltham, MA, USA). The regions of interest (HC and ST) were identified using a stereotaxic atlas,^27^ and the corresponding images were captured using a Leitz Aristoplan microscope coupled to a CCD-72S camera (Dage-MTI, Michigan City, IN, USA). To minimize variability, we used an optical density ratio between the regions of interest and the background for each section and time point. Five randomly selected fields per section from each animal were quantified (ImageJ 4.37, NIH, Bethesda, MD, USA).

For immunofluorescence, the brain sections were incubated with MAX antibody (12 h, RT, in 5% normal goat serum), washed and incubated with TRITC-labeled IgG (1:100; Jackson Labs) for 2 h. The sections were counterstained, mounted with coverslips using Vectashield (Vector Labs) containing DAPI (Prolong Gold antifade reagent, Invitrogen, Carlsbad, CA, USA), and examined using a Leitz Aristoplan microscope.

### Reverse transcription and qPCR

Frozen samples were immersed in TRIzol (Invitrogen) and homogenized (Polytron PT10/35, Brinkmann, NY, USA). Total RNA was isolated according to the manufacturer's instructions, quantified via spectrophotometry (NanoDrop, Thermo Fisher Scientific, Waltham, MA, USA), and verified for integrity on 1% agarose gels. The total RNAs (2 μg) from both groups were simultaneously reverse-transcribed using oligo(dT) primers and SuperScript III (Invitrogen) in a final volume of 20 μl. Quantitative analyses of *Max* and the control genes were performed in a Rotor-Gene 3000 (Corbett Research, Concord, NSW, Australia), as previously described.^21^ Optimal conditions were obtained using a five-point, two-fold dilution curve for each transcript. Each PCR contained 12.5 ng of reverse-transcribed RNA, 200 nmol of each specific primer and SYBR Green PCR Master Mix (Applied Biosystems, Foster City CA, USA). Complementary DNA samples from both groups were assayed in triplicate in the same run. Samples without complementary DNA and with RNA (no reverse transcription) were included as negative controls. A dissociation curve was performed to confirm product specificity and the absence of primer dimers. The relative amount of *Max* transcript in each brain area was calculated as previously described.^28, 29^ The following genes were analyzed as candidate controls: *Ppia*, *Hprt1*, *Gadph*, and *Actb*. Primer sequences were as follows: *Ppia* 5′-AATGCTGGACCAAACACAAA-3′, 5′-CCTTCTTTCACCTTCCCAAA-3′ *Hprt1* 5′-TGTTGTTGGATATGCCCTTG-3′, 5′-GCGCTCATCTTAGGCTTTGT-3′ *Gadph* 5′-AGGAGCGAGACCCCACTAAC-3′, 5′-GTGGTTCACACCCATCACAA-3′ *Actb* 5′-GTGGGAATGGGTCAGAAGG-3′, 5′-GGTCATCTTTTCACGGTTGG-3′ and *Max* 5′-GCAGTGAGGTGGTTGTCGCCC-3′, 5′-ACCTCGGTTGCTCTTCGTCGC-3′.

### Proteasome assay

Frozen brain samples (PFC, HC and ST) were assayed for 20S chymotrypsin-like activity by monitoring the fluorescence intensity of 7-amino-4-methylcoumarin (AMC) after cleavage from the labeled substrate Suc–Leu–Leu–Val–Tyr–AMC (Kit APT280, Chemicon, Temecula, CA, USA). Briefly, all samples were homogenized in lysis buffer (50 mm HEPES, pH 7.5, 5 mm EDTA, 150 mm NaCl, 1% Triton X-100, and 2 mm ATP) and then centrifuged (20 000 *g* for 20 min at 4°C), and protein concentrations were determined using the bicinchoninic acid method (Pierce). Equal amounts of protein (40 μg) were incubated (2 h, 37 °C) in 100 μl of assay buffer containing 10 μl of the supplied proteasome substrate. The proteasome inhibitor lactacystin (10 μl) was used to monitor the specificity of the assay. Free AMC fluorescence was measured at 440 nm (excitation at 380 nm) using an AMC standard curve (0.04–12.5 μm). The proteasome activity in the experimental samples was calculated as the percentage of the controls (in arbitrary units, a.u.).

### Statistical analysis

Student's *t*-tests were used to analyze the differences between the control and experimental groups, with significance set at *P*⩽0.05. Linear regression analysis was performed on the MAX and MYC protein levels in the individual samples. The data are presented as the means±s.e.m.

## Results

### Behavioral testing

Locomotor activity, reported as the distance traveled (2652±220 cm control, 2261±106 cm defeated; *P*>0.05) and time traveled (*P*>0.05; Figure 1b) in the total area of the OF, was not different between groups. Anxiety-related behaviors were investigated using both the OF and EPM. Time traveled in the peripheral area (PA) or central area (CA) of the OF was not different between groups (*P*>0.05; Figure 1b). Similarly, in the EPM, the percentage of time spent in the open arms was not different (*P*>0.05; Figure 1c), indicating that adolescent defeated mice do not show anxiety-like behaviors when tested in either apparatus. In the FST, the defeated mice remained in an immobile or floating position longer than the controls (*t*_(31)_=2.39; *P*<0.05; Figure 1d).

The preference for 2% sucrose on the twenty-first day, an indicator of anhedonia, is reduced in the defeated mice (sucrose intake=1.80 to 7.90 ml; CV=29%) compared with the controls (3.10 to 9.00 ml; CV=20% *t*_(31)_=2.10; *P*<0.05; Figure 1e), whereas the animals maintained a similar total fluid intake (7.96±1.77 and 7.73±2.13 ml, respectively; *P*>0.05). The results of the FS and SP suggest that repeated episodes of social defeat induce a depression-like state in adolescent mice and that this effect is not exhibited by a few extremely stressed animals. Notably, we used this same defeat protocol in adolescent mice that were not tested in the FS before the SP measurements. They also displayed reduced SP compared with the non-defeated mice (data not shown; *P*<0.05), ruling out a possible effect of the acute stress from the FS on the SP results.

### Blood corticosterone levels

The blood CORT concentration at 24 h after the last episode of social confrontation did not differ between control and defeated groups (78.30±39.71 vs 78.05±34.18 ng ml^−1^; *n*=6 each; *P*>0.05).

### MAX and MYC protein levels

**Figure 2:**
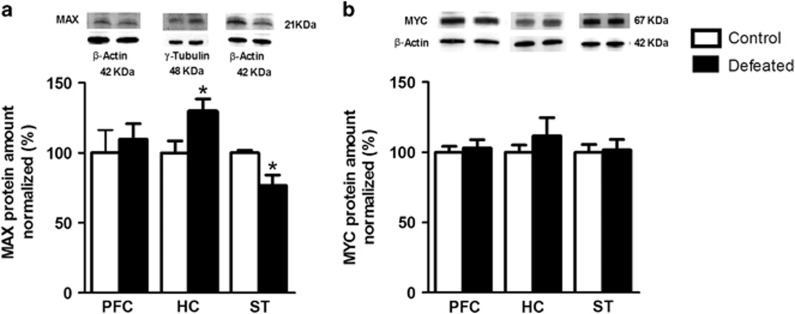
(**a**) Immunoblotting of MAX and (**b**) MYC in the prefrontal cortex (PFC), hippocampus (HC) and striatum (ST) of adolescent male mice submitted to repeated episodes of social defeat over 21 days. The values are expressed as the % of MAX or MYC levels in the control group normalized to β-actin or γ-tubulin (optical density (OD)). Representative images of the MAX and MYC proteins and respective controls are shown. Mean±s.e.m.; *P<0.05; *n*=6–9 each for PFC and HC; *n*=4–5 each for ST.

Compared with the controls, MAX immunoblots from the defeated adolescents show a 30% increase in the HC (*t*_(13)_=2.26; *P*<0.05) and a 20% decrease in the ST (*t*_(7)_=2.71; *P*<0.05; Figure 2a). The MAX level in the PFC is similar between groups (*P*>0.05; Figure 2a), as are the MYC levels in the PFC, HC and ST (*P*>0.05, Figure 2b). Regression analyses of the MAX and MYC levels were assessed separately for each group. A positive correlation between the MAX and MYC levels was observed in the PFC for both the control (*r*=0.92, *P*<0.05) and defeated mice (*r*=0.84, *P*<0.05). However, social stress disrupts this correlation in the HC (control: *r*=0.83, *P*<0.05; defeated: *r*=0.29, *P*>0.05) and ST (control: *r*=0.88, *P*<0.05; defeated: *r*=0.16, *P*>0.05).

**Figure 3:**
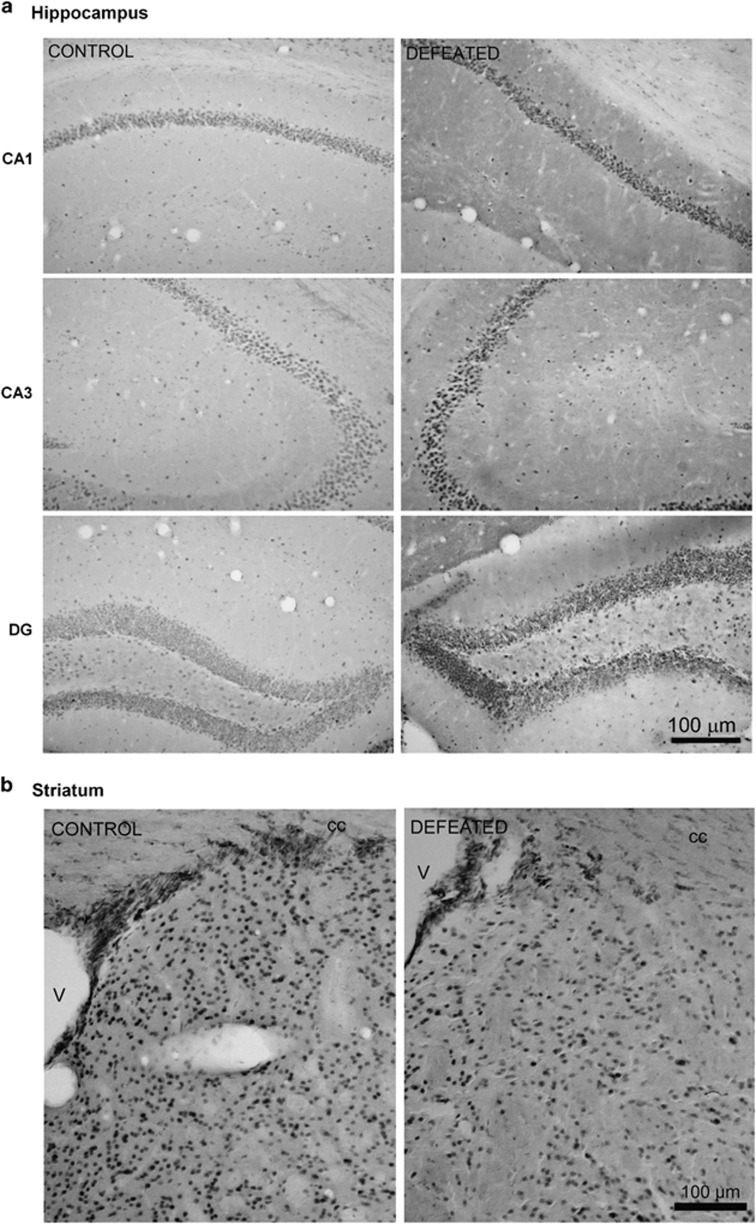
Representative immunohistochemistry images. (**a**) The number of MAX-immunoreactive cells is increased in coronal sections of the hippocampus (CA1, CA3 and dentate gyrus: DG) and (**b**) reduced in the striatum of adolescent male mice submitted to repeated episodes of social defeat over 21 days (v: ventricle, cc: *corpus callosum*; *n*=5 each).

MAX immunoreactivity was consistent with the immunoblotting results. In the HC, the number of MAX-positive cells increased by 36% in CA1 (control: 100±4.4%, defeated: 135.5±9.8% *t*_(8)_=2.32; *P*<0.05), by 29% in CA3 (100±3.0% vs 129.2±8.9% *t*_(8)_=2.45; *P*<0.05), and by 27% in the dentate gyrus (DG; 100±8.2% vs defeated: 127.3±3.2% *t*_(8)_=2.90; *P*<0.01; Figure 3a). In the ST, the number of MAX-immunoreactive cells decreased by ~89% in the defeated mice (control: 100±29.0%, defeated: 10.64±49.4% *t*_(8)_=3.03; *P*=0.01; Figure 3b).

**Figure 4:**
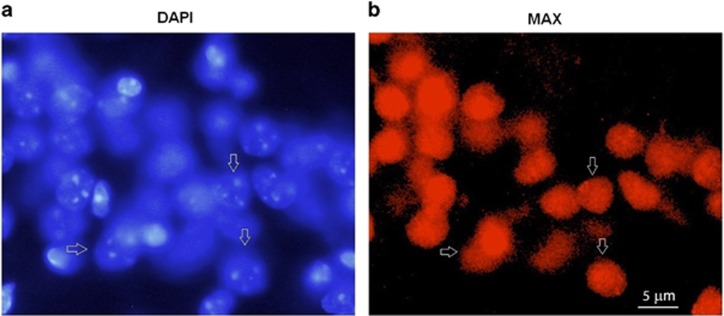
Nuclear expression of MAX protein in the hippocampus of C57BL/6 adolescent male mice. (**a**) Nuclei were stained with DAPI (blue). (**b**) MAX fluorescence (red). Arrows indicate nuclei positive for MAX protein.

To further characterize the subcellular localization of MAX in the HC, slices were stained with the nuclear marker 4′,6-diamidino-2-phenylindole (DAPI) and anti-MAX antibody. The MAX signals were mainly detected in the nuclei of the hippocampal cells (Figure 4).

### MAX transcript levels

**Figure 5:**
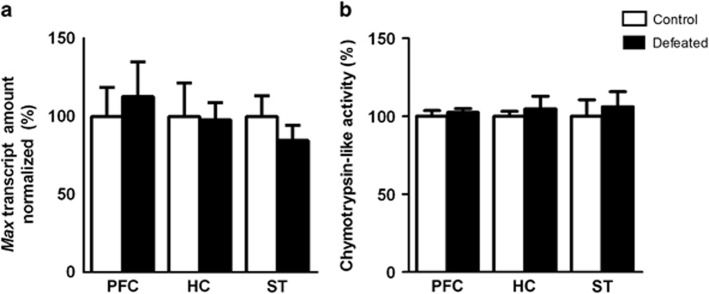
(**a**) *Max* transcripts normalized to the *Ppia* levels (*n*=6 each) and (**b**) chymotrypsin-like activity of the 20S proteasome in the prefrontal cortex (PFC), hippocampus (HC) and striatum (ST) of adolescent male mice submitted to repeated episodes of social defeat over 21 days (*n*=5 each). The values are expressed as the percentage of the control group. Mean±s.e.m.; *P*>0.05.

The *Max* transcript levels in the PFC, HC, and ST of the defeated mice were similar to the controls (*P*>0.05 for all; Figure 5a). Transcript levels for all tested control genes were similar between groups in the HC (*Ppia*: 0.63±0.11, 0.79±0.19; *Hprt1*: 0.34±0.16, 0.40±0.03; *Gapdh*: 0.74±0.19, 0.88±0.16; *Actb* 0.49±0.28, 0.56±0.17 for controls vs defeated, respectively), PFC and ST (not shown) (*P*>0.05 for all). *Ppia* showed the most stable levels across samples and was used to normalize the *Max* levels.

### Proteasome activity

We measured the relative chymotrypsin-like activities of the two groups to determine whether the changes observed in the protein but not transcript levels of MAX in the defeated mice were associated with dysfunction of the proteasome. The enzyme activity (a.u.) was similar between groups in all three brain areas (*P*>0.05; Figure 5b).

## Discussion

Using repeated brief episodes of social defeat over 21 days as a stressor and maintaining the defeated mice in social groups after each defeat, we show that male adolescent mice exhibit a depression-like phenotype, as indicated by reduced sucrose preference (an anhedonic response) and prolonged immobility in the FST. These behavioral changes are not accompanied by differences in spontaneous motor activity or anxiety-like behaviors in the OF or EPM. Importantly, we also show that repeated social defeat is associated with region-specific differences in the protein levels of the transcription factor MAX, but not in its transcript, in the hippocampus and striatum. Proteasome activities in these brain areas are not modified by the social stress. The transcription factor MYC, a known partner in the MAX network, is not differentially expressed in these brain areas following social defeat.

### Repeated social defeat stress associated with group housing induces depression but not anxiety-like behaviors in adolescent male mice

Social defeat is an ethologically relevant animal model of psychosocial stress, and although it can be used as a model of adolescent bullying, most studies have been conducted on adult animals. Only recently have there been reports on mice that were socially defeated and evaluated during adolescence. Two laboratories have used the 10-day protocol in male adolescent C57BL/6 mice and have shown increases in both anxiety and depression-like behaviors.^30, 31^ In these two studies, the intensity of physical and psychological aggression received by the adolescents was much higher than that used in our protocol. Indeed, Huang's study reported elevated levels of mortality among the adolescent mice after the social defeat (~21.3%).^30^ In our protocol, although the social defeat occurs over a longer time (21days), the adolescents were removed from the physical interaction as soon as they displayed submissive postures (up to 5 min), and the threat period lasted only 30 min per day. We have never observed any mortality or physical incapacities among our defeated adolescents.

The other important difference with our procedure is that after this shorter exposure to stress, the adolescents are returned to their original cages, where they are regrouped with their familiar cagemates. Actually, we have used this protocol as a prospective method that is more similar to bullying episodes occurring among boys in their social environments. In contrast, in the 10-day protocol, defeated mice never return to their home cages.^32, 33^ In adolescent mice, social isolation *per se*, including the prevention of social play behaviors, induces detrimental effects including molecular changes in the serotonergic system, particularly in emotion-related brain areas.^20^ The housing conditions after social defeat are crucial to the magnitude and duration of the effects of stress.^10, 34^ A reduction of stress responses by living with conspecifics is termed ‘social buffering'.^35, 36^ Accordingly, the anxiety-like behaviors in an EPM after social defeat are lower in group-housed adult rats than in individually housed adult rats.^37, 38^ These studies in rats indicate that social housing following social defeat attenuates and/or prevents increases in anxiety-like behaviors. Although our protocol does not include an experimental group housed in social isolation, our results in adolescent mice that were socially housed after each defeat are consistent with these findings because the two independent cohorts of mice did not differ from the undefeated controls in parameters related to unconditioned anxiety. Dissociation of spontaneous (non-social) anxiety-like behaviors and the hedonic state after exposure to stressors was previously reported in adult mice.^39, 40^ Those studies used mice subjected to isolation housing and observed increased anxiety after chronic stress, which was independent of the differences in the SP. These results may indicate that elevated anxiety is a non-specific and common response to the severity of chronic stress and is not related to the development of anhedonia.

Although the milder conditions used in our current study are not sufficient to induce anxiety-like behaviors, they are sufficient to induce anhedonia in the adolescents. The decreased intake and preference for palatable solutions in rodents has been used to indicate a presence of anhedonia, a core feature of depressive disorder in humans.^41, 42^ Studies using social defeat have consistently shown decreased SP,^39, 40, 43, 44^ suggesting a depression-like behavior as a consequence of this stress.

An additional behavioral alteration indicative of depression in our socially defeated adolescents is the increased immobility time in the FST. This test is widely used to assess antidepressant efficacy.^45^ Our adolescent defeated mice show a more passive coping strategy when exposed to the acute stress of swimming than do the non-defeated mice. This result is consistent with recent reports using the 10-day social defeat protocol in mice.^30, 31^

The hypothalamic-pituitary-adrenal axis is involved in stress adaptation, and glucocorticoids coordinate physiological processes in response to stress. Because our goal was to determine whether these animals would exhibit sustained corticosteronemia when the brain samples were collected, we measured the blood CORT concentrations 24 h after the last episode of social defeat. Regardless of whether they were subjected to social defeat stress, 52-day-old mice did not display differences in the circulating CORT levels at this single time-point. It should be noted that a steady-state CORT determination was performed in resting animals 2–3 h before the lights were turned off, without any additional acute stress to challenge the hypothalamic-pituitary-adrenal axis. These findings are consistent with the observation that many individuals with depressive symptoms do not experience basal hypercortisolemia.^46, 47^ Regarding the role of corticosteroids in stress, studies have shown that their levels are increased in acute situations but are not modified after chronic mild stress,^48, 49^ suggesting that hormone levels are adapted after exposure to long-lasting stress.^46^

Taken together, these results show that this protocol of repeated brief social defeat in adolescent mice associated with group housing can also serve as an interesting animal model of a subtype of depression that is dissociated from generalized anxiety.

### Social stress in adolescents induces region-specific alterations in the MAX protein levels

We studied MAX expression in selected brain areas known to be involved in emotional behaviors, the HC and PFC, and displaying the highest expression in the neonatal mice cerebrum.^14^ Repeated defeat stress induces a significant increase in MAX protein expression in the HC, particularly in CA1, CA3 and the DG, but does not change MAX expression in the PFC. The selective increase in the MAX levels in the HC may reflect a neuroprotective response to the social stress because this area remains structurally plastic throughout life.^50^ Interestingly, a careful inspection of the reported steady-state MAX expression in mice (see Table 4 from ref. 14) suggests that the signal intensity increases from the infancy to adult periods, specifically in the DG. This result is consistent with a role for MAX in cell proliferation. Accordingly, a protective role for MAX overexpression in cultured rat endothelial cells has been reported.^51^

Unexpectedly, the MAX level is severely reduced in the ST. The dorsal striatum collected here comprises the caudate and putamen and was chosen as a "negative control" area because it is not as closely associated with emotional behaviors as is the ventral "affective" striatum. However, recent studies have challenged this notion, supporting alterations in primary functional connectivity involving dorsal but not ventral corticostriatal circuits.^52, 53^ In particular, in depressed adolescent populations, an elegant imaging study showed that alterations in dorsal striatum connectivity are evident at the early stages of illness, suggesting compensatory mechanisms.^54^ Therefore, our findings of perturbed expression of MAX in the dorsal striatum of adolescent male mice displaying depression-like behaviors may further indicate a role for this brain region, although the participation of MAX in this process remains unspecified.

### MAX is localized in most nuclei from hippocampal cells

MAX-immunopositive signals in the hippocampal cells of the defeated mice are detected homogeneously but only in the nucleus. This result is consistent with previous studies showing that MAX protein is expressed in a homogeneous nuclear pattern.^55, 56^ A closer inspection of the MAX-immunolabeled cells suggests that they are indeed neurons and not glial cells and that MAX is present in most of the DAPI-labeled nuclei. This neuronal sub-localization of MAX is consistent with its role as a transcription factor.

### The social defeat-induced differences in MAX protein levels are not related to its gene expression or protein degradation

Interestingly, the perturbed MAX levels observed in the HC and ST of the defeated adolescents, as supported by two immunoassays, do not seem to reflect differential gene transcription. The quantitation of the *Max* transcripts shows similar levels between groups in all brain areas studied. Intracellular proteolysis in eukaryotic cells mainly occurs via the ubiquitin–proteasome system, and its dysregulation has been reported in several central nervous system pathologies, including neurodegenerative diseases and autism spectrum disorders.^57^ Genetic studies have associated polymorphisms in ubiquitin-proteasome system-related genes with major depression,^58^ antidepressant responses^59^ and generalized anxiety disorder.^60^ Because the intranuclear MAX levels can be modulated by proteasomal degradation *in vitro*,^61^ we hypothesized that a dysfunction of this system could account for our results. However, for all brain areas analyzed, the chymotrypsin-like activity of the proteasome was similar between the defeated and control mice. These results do not support the hypothesis that the differences in MAX protein expression in the defeated mice result from perturbed degradation by this system in response to social stress. Another possibility would be differential regulation of the translation of *Max* mRNA. Accordingly, brain region-specific interference in the translational control was reported in response to chronic mild stress^62^ and after chronic fluoxetine administration in rats.^63^ *Max* is also directly targeted and repressed by the miR-22,^64, 65^ a microRNA recently implicated in the pathogenesis of psychiatry disorders.^66, 67^ Blood miR-22 is upregulated by chronic antidepressant treatment in depressed subjects,^68^ being therefore suggested as a molecular signature. These interesting hypotheses deserve investigation in the adolescent social defeat stress model.

### MYC does not correlate with the MAX disturbances induced by social defeat

The MAX network comprises a group of transcription factors that act by dimerization through their helix-loop-helix zipper domains.^12^ Transcriptional activation is mediated exclusively by MAX-MYC, whereas other complexes may mediate transcriptional repression.^69^ Unlike MAX, which is a constitutive protein, MYC has a short half-life and is highly regulated.^70^ MYC is thought to influence up to 15% of genes, and despite their broad functional range, MYC affects specific classes that are involved in metabolism, protein biosynthesis, cell cycle regulation, cell adhesion and the cytoskeleton.^71^ The *c-Myc* and *Max* genes show similar temporal expression patterns in the brain, that is, higher levels during embryonic and neonatal stages.^72^ Therefore, we hypothesized that MYC may also be an important MAX partner, and its expression would be similarly disturbed by social stress in adolescence. However, immunoblots do not reveal differences in the MYC levels between the defeated and control mice in any brain area, which argues against this possibility. Furthermore, we show a positive linear association between the MAX and MYC protein levels in the three brain areas of the control adolescents; this correlation is disrupted in the HC and ST of the defeated mice, where MAX is modulated, but not in the PFC, where MAX levels remain unchanged.

Although social defeat does not alter the MYC levels, it remains possible that the disturbed MAX/MYC ratio in the HC and ST indirectly affects its transcriptional activity. Accordingly, *in vitro* studies suggest that overexpression of MAX in the absence of a corresponding increase in MYC can affect MYC function because MAX homodimers, which are transcriptionally inert, compete for binding to the same specific DNA elements.^15, 73, 74^ It is not clear what factors determine the identities of their direct targets because it also depends on the specific transcriptional cofactors recruited and their chromatin-modifying activities.^15^

### A new biological role for MAX?

The complexity of the MAX network of transcription factors is most frequently explored in tumorigenesis. A recent human study identified MAX mutations as a cause of hereditary pheochromocytoma, a tumor with neuroendocrine features, suggesting a role for MAX as a classic tumor-suppressor gene.^75^ Max was found to be inactivated in small-cell lung cancer,^76^ and tricyclic antidepressants inhibit the small-cell lung cancer and other neuroendocrine tumors both *in vitro* and in animal models.^77^ In non-tumor cells, normal expression of MAX network components drives embryonic development and tissue repair,^78^ but the specific physiological importance of MAX in various brain areas or in neuroplasticity and stimulus regulation is unknown. MAX, but not MYC, is downregulated in the *postmortem* dorsolateral PFC from schizophrenia patients, being therefore considered a potential biomarker for this disease.^79^ Our results indicate that MAX, but not MYC, is differently expressed in brain areas involved in depression-related behaviors in adolescent mice after social stress. Because interactions with alternative partners can influence both the activity and target genes of transcription factors, it will be important to identify the associated partners, if any, and the dynamics of the MAX interactions in these areas. What candidate genes are regulated by MAX in our model? Can social stress in adulthood also modulate MAX? Can chronic antidepressant treatment normalize behavioral deficits and MAX levels? Among several others, the main question is the functional importance of this region-specific modulation in depressive behavior. Although our findings are correlational, we speculate that the increased MAX levels in the HC, a brain area with extensive structural plasticity, would reflect a neuroprotective response to social stress in adolescent animals. This hypothesis is currently under investigation.

In conclusion, adolescent male mice show depression-like behaviors but not anxiety-like behaviors after repeated brief social defeat stress with group-housing conditions between each stress episode. These mice display elevated expression of the transcription factor MAX in the nuclei of most hippocampal neuronal cells, reduced MAX expression in the dorsal striatum and similar levels in the prefrontal cortex. These changes in MAX protein levels are not mediated by its transcript levels or proteasomal degradation. Furthermore, changes in brain MAX levels are not reproduced by its putative dimerization partner MYC, suggesting a dissociated or independent role. Our findings provide novel insights into the molecular mechanisms related to depression in adolescence. Although the MAX network is frequently studied in cell behaviors in oncology, our results suggest that psychiatry should pay more attention to involvement of these proteins in the individuals' behaviors.
